# The application of patient-derived organoid in the research of lung cancer

**DOI:** 10.1007/s13402-023-00771-3

**Published:** 2023-01-25

**Authors:** Yin Li, Xinyu Gao, Chao Ni, Bing Zhao, Xinghua Cheng

**Affiliations:** 1 Department of Oncology, Shanghai Chest Hospital, Shanghai Jiao Tong University School of Medicine, Shanghai, China; 2 State Key Laboratory of Genetic Engineering, School of Life Sciences, Fudan University, Shanghai, China; 3 Institute of Organoid Technology, bioGenous Biotechnology, Inc, Suzhou, China

**Keywords:** Patient-derived organoid, Lung cancer, Co-culture, Personalized therapy

## Abstract

Lung cancer is the most common cancer and the leading cause of cancer-related death worldwide. However, mechanisms of its progression remained unclear and new treatments against this disease are rapidly emerging. As a novel preclinical model, patient-derived organoid (PDO) can also be established from the patient’s tumor tissue and cultured in the laboratory, which preserves the key biological characteristics of the original tumor. Compared to the patient-derived xenograft (PDX) model of lung cancer, the culture success rate is improved, and the time and cost of model establishment are largely reduced. PDO is also expected to provide a more individual model to predict the efficacy of anti-cancer treatment in vitro. This paper summarizes the current application of PDO in the translational research of lung cancer.

## Introduction

Lung cancer is the most common cancer worldwide. It is also the leading cause of cancer related death (1.8 million deaths each year) [1]. Lung cancer development is closely associated with genetic alterations such as epidermal growth factor receptor (*EGFR*) mutation, anaplastic lymphoma kinase (*ALK*) rearrangement, and kirsten rats arcomaviral oncogene homolog (*KRAS*) mutation [2], and its progression and metastasis are also associated with microenvironment changes. Although new drugs that targeting the key molecular events may impede or even reverse this process, their effects remain hardly predictable and all develop drug resistance eventually. Therefore, to promote drug screening and translational research of lung cancer an in vitro model which preserves the biological characteristics of the original tumor is urged.

Consistence with patient-derived xenograft (PDX), tumor organoids reflect the phenotype and genetic characteristics of the original tumor with higher fidelity compared to cancer cell lines [3]. Meanwhile, the culture success rate and culture period are significantly improved as against to the PDX, which shows the potential of organoids to complement existing model systems [4, 5]. Tumor organoids can be derived from murine tumor cells or induced pluripotent stem cells (iPSC), and more importantly from fresh patients tumor tissue, named as patient-dived organoid (PDO) [6]. The PDO formation is more efficient in the way of omitting animal modeling or cell differentiation, which facilitates the efficient clinical translation of organoid technology [7]. In addition, PDO provide a broad spectrum of lung cancer stage including precancerous lesions or early-stage tumors. This novel system closely relevant to primitive tumor thus provide valuable information for tumor biology, drug development, patient responsiveness prediction, and clinical transformation guidance in a high-throughput approach [8–10]. Although PDO still exist problems such as tumor cell purity and inevitably heterogeneity lost, modified culture system and advanced technology integrate may be helpful [9, 11–13]. In this review, we elaborate on the current role and potential clinical applications of PDO in lung cancer research and discuss its limitations and future expectations.

## PDO reproduces the biological characteristics of lung cancer in vitro

### PDO as a preclinical model for lung cancer

Lung cancer PDO has some advantages over other preclinical models, comparing to traditional cancer cell lines and the PDX model (Table 1).

**Table 1:** Comparison of preclinical models for lung cancer

Model	Cell line	PDX	PDO
Time consumption	+	+++	+
Cost	+	+++	++
Sample acquisition	+	+++	++
Success rate	+++	+	+++
Matched normal tissue	-	-	+++
Genetic modification	+++	-	+++
Genetic background retention	-	+++	+++
Tumor heterogeneity	-	++	+
Drug efficacy prediction	-	++	++
TME interaction research	-	+	+^*^
Immunotherapy evaluation	-	+	++^*^
High-throughput drug testing	+++	-	++
Biobanking	-	-	+++
Personalized therapy	-	++	++

PDX: patient-derived xenograft, PDO: patient-derived organoid, TME: tumor microenvironment, +: low correlation, ++: medium correlation, +++: high correlation, -: not suitable, *: Based on co-culture conditions for organoids

Cell lines remains the most used in vitro model in cancer research. However, lung cancer cell lines also have some significant limitations. Firstly, it does not reflect individual tumor characteristics real-time and the success rate of establishment from primary lung cancer is less than 5% [14]. Secondly, random genetic drift caused by long-term culturing and repetitive passaging produces artifacts cannot reflect the genetic background and individual epigenetic differences of lung cancer [15]. In addition, cancer cells cultured in three dimensions are more close to physiological state in vivo differentiating them from those monolayer cultured cell lines [16]. Furthermore, experimental therapies using established cell lines for preclinical testing has a high failure rate in phase III clinical trials, which indicates its inherited application defects in tumor research and drug development [17, 18].

Lung cancer PDX mice model, as compared to tumor cell lines, retains more genomic and phenotypic characteristics of the primary tumor. Drug testing results based on PDX models reproduce the clinical outcomes, thus it is considered a potential preclinical model for translational research and personalized drug screening [19]. Transplantation of human leukocytes and purified CD34 (+) hematopoietic stem cells into immunodeficient mice may mimics the functional human immune system, allowing PDX to be used in vivo to assess the therapeutic response of lung cancer cells to Pembrolizumab and Nivolumab [20]. However, to establish a successful PDX model is time consuming and costly, thus significantly limiting its application in large-scale drug screening and personalized drug guidance [21]. Although PDX provides complete tumor environment for cancer cell growth, transplanted human stromal cells have tendency to be replaced by murine counterparts. Species-specific cytokines may cause drastic changes in drug responses [19, 22]. Besides, genetic alterations and chromosome abrasions are also inevitable in PDX passaging [21].

PDO summarizes histopathology, gene expression profile, and treatment sensitivity of the primary tumor [23]. PDOs derived from different lung cancer patients show different morphology under hematoxylin-eosin (HE) staining but maintain similar morphology with the primary tumor tissues. HE staining and immunohistochemical is also used to identify the purity of tumor cells in lung cancer PDO [5, 23–26]. According to the whole-exon sequencing, whole-genome sequencing and RNA-seq of lung cancer PDO, Kim and their colleagues reported that short-term cultured lung organoids have retained 92.7 and 77% of the driver mutations in the primary tissue, respectively [4, 27]. Notably, some PDOs harbored additional driver mutations that were not detected in the match tissue may related to cross-cell contamination, limitations of genetic tests or low frequencies in the original tumors. Compared to short-term cultured PDO, long term passage (passage > 10) PDO maintain the overall mutation spectrum and copy number variation detected in the original tissues, although 80% of long-term cultured PDO have increased mutation numbers, suggesting the sub-clonal expansion [4, 5, 23, 25, 27].

### Lung cancer PDO in the research of oncogenesis

Metabolic reprogramming is one of the major steps of oncogenesis [28]. Glutamine synthetase (GS) is overexpressed in cancer and promotes cancer cell growth through glutamine anabolic metabolism [29]. Knockdown of GS in lung cancer PDO can inhibit organoid growth. Depletion of GS also restores the sensitivity of PDO to the microtubule drug Paclitaxel [30]. Using lung cancer PDO, Chen et al. and their colleagues showed reactive oxygen species (ROS) play pivotal roles in epithelial-mesenchymal transition and cell invasion and migration. Fangchinoline, a small molecule drug, revise this malignant progression by reducing cytoplasmic ROS and inhibit the Akt-mTOR signaling pathway [31]. It was also found that NF-κB and MYC were overexpressed in CD133 (+) CD44 (+) lung cancer PDO, and treatments targeting these signaling pathways may be is a possible treatment for the patients [32]. High invasiveness of CD133 (+) colonies in lung cancer PDO are also associated with activation of *AXL*, *TGFβ*, and *JAK1*, proofed by effective treatment of AXL inhibitor TP-0903 and JAK inhibitor Ruxolitinib [33].

Besides genomic research, lung cancer PDO can be also used to explore the epigenetic changes of lung cancer. Sca-1 (+) CD24 (+) double-positive lung adenocarcinoma (LUAD) cells are a subtype with high tendency of proliferation and invasion. Using LUAD PDO, Rowbotham et al. found that this aggressive phenotype is controlled by H3K9 methyltransferases G9a, a suppressor of tumor-propagating cell phenotype in lung cancer cells. For specific individuals with this subtype, demethylase inhibitors rather than methylase inhibitors contribute to the treatment of advanced lung cancer [34].

### The diversity of sample sources for lung cancer PDO

Various research groups have reported the successful establishment of lung cancer PDO from different sources (Table 2). Compared to PDX and cancer cell line, lung cancer research using PDO is at a climbing stage [35]. Besides surgically resected tumor, PDO can also be cultured from malignant pleural effusion or biopsy tissue. Although the success rate remains to be improved (the current success rate is approximately from 28 to 83%) [4, 9, 11, 36, 37].

**Table 2:** Successfully established PDOs in lung cancer and their application

Sample source	SR	Tumor clinical stage/No.		Application	References	
		I stage	>I stage			
6 LUAD tissue, 77 pleural effusions	Overall 83%	0	83	Biobanking	[4]	
16 LUAD tissue, 14 LUSC tissue	84.2%, 93.3%	17	17	Biobanking	[23]	
18 NSCLC biopsy, 16 NSCLC tissue	28%, 88%	-	-	Biobanking	[36]	
12 LUAD tissue	80%	4	8	Biobanking	[5]	
10 SCLC tissue	-	-	-	Biobanking	[25]	
12 LUAD tissue, 6 LUSC tissue, 1 SCLC tissue	-	-	-	Biobanking	[27]	
6 NSCLC tissue, 4 NSCLC biopsy	Overall 17%	0	10	Biobanking	[11]	
2 LUAD tissue	-	-	-	Tumor formation and progression research	[34]	
3 LUAD tissue, 3 LUSC tissue	-	-	-	Tumor formation and progression research	[32]	
2 LUAD tissue	-	-	-	Tumor formation and progression research	[31]	
6 LUAD tissue	60%	5	1	Tumor formation and progression research	[33]	
14 LUAD tissue	-	8	6	Drug resistance exploration	[57]	
2 SCLC tissue	-	-	-	Drug resistance exploration	[62]	
2 LUSC tissue	-	0	2	Drug resistance exploration	[61]	
5 LUAD tissue, 1 LUSC tissue	-	2	4	Drug resistance exploration	[60]	
LUAD tissue	-	-	-	Drug resistance exploration	[58]	
1 LUAD biopsy	-	0	1	Prospective targeted therapy	[37]	
1 LUAD tissue	-	1	0	Prospective targeted therapy	[63]	
1 LUAD biopsy, 1 pleural effusion	-	-	-	Prospective targeted therapy	[64]	
2 NSCLC pleural effusion	-	-	-	Prospective targeted therapy	[65]	
2 LUAD tissue	-	-	-	For drug screening	[67]	
2 NSCLC tissue	-	-	-	For drug screening	[66]	
55 LUAD tissue, 18 LUSC tissue, 4 SCLC tissue, 3NSCLC biopsy	77.5%, 78.3%, 100%, 37.5%	27	50	For drug screening	[9]	
10 NSCLC tissue	71.4%	2	8	For drug screening	[68]	
6 LUAD tissue, 1 LUSC tissue	-	3	4	For drug screening	[26]	
2 NSCLC tissue	-	-	-	For drug screening	[30]	
SCLC tissue	-	-	-	For drug screening	[69]	
6 LUAD tissue	-	2	4	For TME interaction	[44]	
1 LUAD tissue, 2 LUAD biopsy, 2 LUSC tissue	Overall 83.3%	2	3	Immunotherapy evaluation	[13]	
4 LUAD tissue, 3 LUSC tissue	57.1%, 75%	1	6	Immunotherapy evaluation	[12]	
14 LUAD tissue, 6 LUSC tissue	87.5%, 85.7%	7	13	Immunotherapy evaluation	[54]	
1 LUAD tissue	-	-	-	Immunotherapy evaluation	[55]	
1 LUAD tissue, 2 LUSC tissue	-	-	-	Immunotherapy evaluation	[10]	
2 LUAD tissue, 1 LUSC tissue	-	-	-	Immunotherapy evaluation	[53]	

PDO: patient-derived organoid, LUAD: lung adenocarcinoma, LUSC: lung squamous cell carcinoma, NSCLC: non-small cell lung cancer, SCLC: small cell lung cancer, TME: tumor microenvironment, SR: success rate

## Co-culture model of lung cancer PDO

### Co-culture PDO model in the research of tumor microenvironment

Changes in tumor microenvironment (TME) affect cancer growth, migration, and invasion, thus are associated with patient prognosis and treatment response [38]. TME includes extracellular matrix (ECM) and stromal cells. There are as many as 52 subtypes of stromal cells in lung cancer, and the subtypes have different proportions among different patients [39]. As mentioned above, cancer cell lines cannot reflect the actual phenotype of the primitive tumor. Although PDX contains a tumor microenvironment, it is difficult to observe and intervene its microenvironment in cancer research. Establishing cell-matrix and cell-cell interactions in tumor PDO may help to understand carcinogenic mechanisms and drug development [40].

**Fig. 1:**
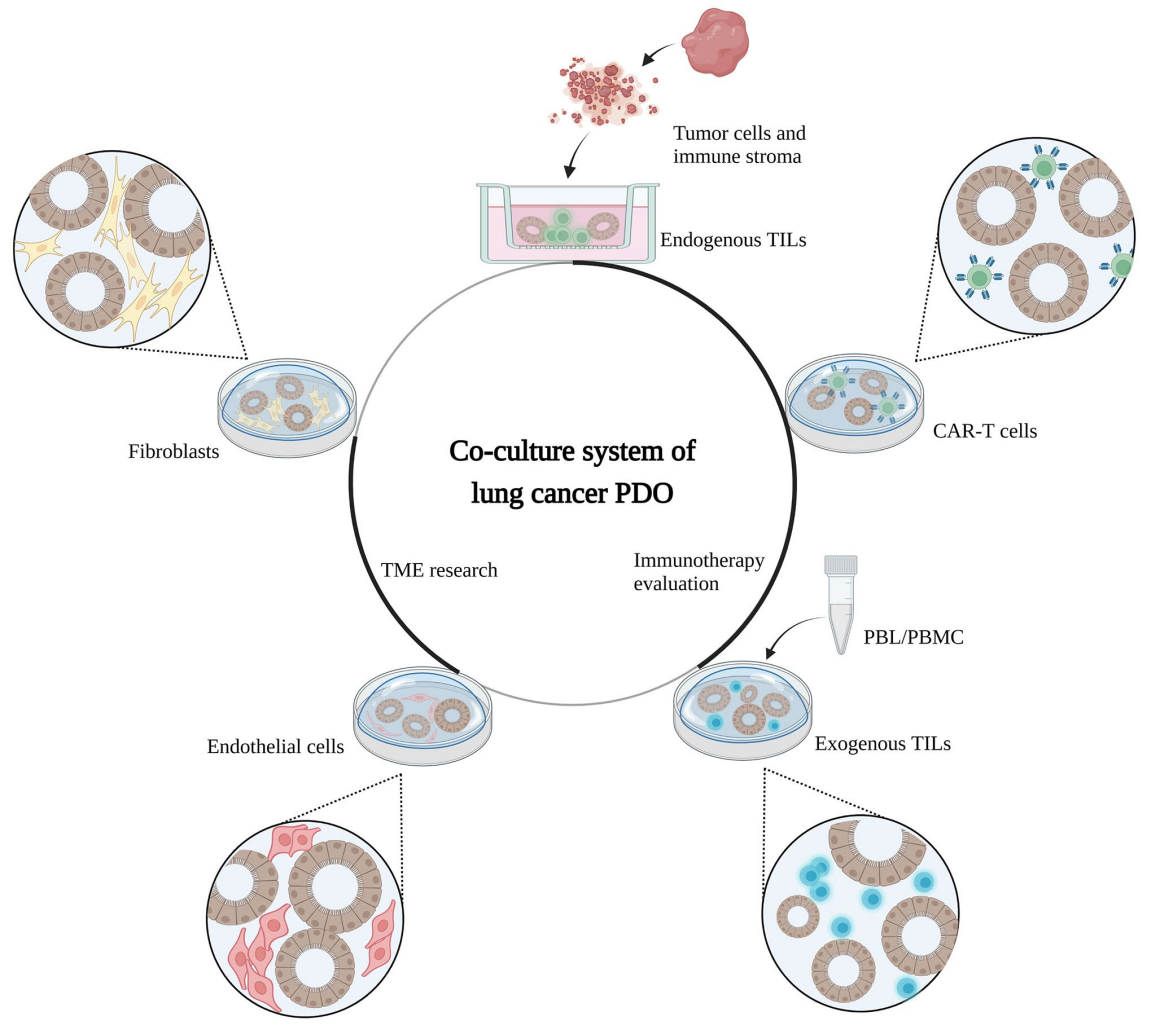
Co-culture system of lung cancer PDO. Co-cultured with fibroblasts or endothelial cells, lung cancer PDO is allowed to research cell interactions in TME. Co-culture system is also suitable for immunotherapy evaluation. Endogenous TILs or exogenous TILs can be generated successfully with the co-culture of original immune stroma and PBL/PBMC correspondingly

Co-culture by supplementing the medium with stromal cells, including endothelial cells, immune cells, and fibroblasts, allows in vitro observation and quantitative study of cell-cell interactions [13, 41–43] (Fig. 1). The specific CD133 (+) stem cell colony presenting in LUAD PDO has a higher proliferation rate due to its excess expression of the Wnt signaling pathway promoter PORCN. Extracellular vesicles secreted by co-cultured peripheral fibroblasts further promote PORCN expression, creating a friendly microenvironment for tumor cell proliferation [44]. Additionally, co-culture of LUSC PDO with carcinoma-associated fibroblast (CAF), which involve tumor progression by actively interaction with other cell types in the tumor microenvironment, enhanced PDO formation [45].

Besides the natural ECM, the emergence of extracellular matrix removal culture has brought a breakthrough for organoid co-culture model. New materials such as composite hydrogels allow the co-culture of PDO and stromal cells, and have steerable biochemical signals and independent mechanical properties changes, making the growth, development, and morphology of PDO more controllable [46]. Using PEG-fibrin hydrogel as a scaffold for PDO, Del Bufalo and their colleagues found that lung cancer cells co-cultured with fibroblast MRC5 grow faster, and the combination of MRC5 and endothelial cells HUVECS in the co-culture system further enhanced tumor cell proliferation and invasion. These results highlight the potential of the co-culture system in the research of TME [47].

### Co-culture and immunotherapy evaluation

Inhibitors of immune checkpoint pathways such as the programmed cell death protein-1/programmed death ligand-1 (PD-1/PD-L1) pathway have shown exciting therapeutic effects in lung cancer recently [48, 49]. However, their effects in individual patient remain largely unpredictable [50]. PDO, along with co-culture system, is assessed as an in vitro and patient-based platform to observe T cell-mediated tumor recognition to help us understand the key factors that determine successful anti-tumor immune responses and to screen suitable therapeutic schedule [13] (Fig. 1).

Recent reports point to the potential feasibility of lung cancer PDO co-culture in immuno-oncology research. The first method is called the reductive simulation method. Researchers hope to generate tumor-reactive T cells by co-culturing matched patients’ peripheral blood lymphocytes (PBL) with tumor cells. The success rate of the tumor-reactive CD8 (+) T cell population is between 33 and 50% hence the co-culture system is a potential platform for evaluating the interaction between tumor cells and T cells in lung cancer PDO [13, 51]. In another co-culture model of peripheral blood mononuclear cell (PBMC) and non-small-cell lung cancer (NSCLC) PDO, MEK-targeted drugs and immune checkpoint inhibitors have synergistic anti-tumor effects by increasing T cell reactivity [12]. xCELLigence is a non-invasive, label-free, real-time cell impedance monitoring technique that evaluates the efficacy of immune checkpoint inhibitors in vitro by assessing cytolysis [52]. Using xCELLigence as a evaluation tool, PD-1 inhibitor Nivolumab or Pembrolizumab alone has no significant effect in lung cancer PDO because of the loss of immune cells retained from the original tumor tissue. In contrast, PD-1 inhibitor causes significant tumor cell lysis in the co-culture system of PBMC and lung cancer PDO, indicating the importance of co-culturing technique for immunotherapy evaluation [10]. Moreover, to increase the immunotherapy testing speed of in vitro platform, Ding et al. developed an automated microfluidic droplet platform that can rapidly generate considerable amount of lung cancer PDO and reliably evaluate the efficacy of PD-1 blockade, bispecific therapy, and T-cell therapy on patients within 7–14 days [53].

Besides the reductive simulation method, another holistic approach is to culture PDO based on an air-liquid interface (ALI), which preserves the original immune components of the tumor, rather than adding additional blood cells to produce endogenous and homologous tumor infiltrating lymphocytes (TILs). This organoid model derived from NSCLC patients preserves the functional and original tumor microenvironment and successfully simulates the immune checkpoint blockade to recover the anti-tumor activity of activated TILs, and the only drawback is that this kind of TIL cannot remain in the medium for more than 60 days [54]. In addition, through the co-culture system, PDOs are also suitable for evaluating the efficacy of chimeric antigen receptor engineered T (CAR-T) cells. CAR-T cells exhibit antitumor activity in LUAD PDO by targeting B7-H3 [55]. We expect an improved and more stable PDO model to demonstrate the feasibility of providing efficacy prediction for precision immunotherapy in future clinical trials.

## Clinical research and drug screening based on lung cancer PDO

In recent years, the use of PDO for individualized drug selection is under clinical investigation [5]. The application of PDO in a large-scale prospective clinical cohort helps to deeply integrate molecular biological characteristics and treatment response in cancer patients, reduce the time of clinic-laboratory-clinic cycle, and establish a medical platform for precision oncology [56]. Thirteen lung cancer PDO-related clinical trials have been registered in the Clinical Trials (Table 3).

**Table 3:** Registered clinical studies of PDO in lung cancer

Register ID	Status	Brief title	Estimated enrollment	Population	Intervention	Outcome	Study design	Nation	Registration date
EUCTR2014-003811-13-NL	Not recruiting	Evaluate the efficacy of patient-derived tumor organoids to successfully allocate patients for treatment with specific targeted agents	70	Patients with locally advanced or metastatic NSCLC	Drug: Palbociclib, PF-04449913, PF-05212384, Inlyta, Selumetinib	ORR	Interventional/Phase2	Netherlands	2016/3/23
NCT03146962	Recruiting	High dose vitamin C intravenous infusion in patients with resectable or metastatic solid tumor malignancies	78	Patients with histologically proven early stage or locally advanced lung cancer (cohort A), Patients with inoperable, metastatic extended RAS or BRAF mutant lung cancer (cohort B)	Drug: vitamin C	3-month DCR (cohort B), change in antitumor activity (cohort A)	Interventional/Phase2	United States	2017/4/27
NCT03453307	Recruiting	Drug sensitivity correlation between patient-derived organoid model and clinical response in NSCLC patients	100	Late-stage NSCLC patients	-	Correlation of ex vivo sensitivity test on patient-derived organoid models	Observational	China	2018/2/27
NCT03655015	Recruiting	Patient-derived organoid model and circulating tumor cells for treatment response of lung cancer	150	Any clinical stage of lung cancer	Procedure: lung tumor resection	Biobank of PDOs, responses of PDOs to chemotherapeutic and targeted agents	Observational	United States	2018/8/29
NCT03778814	Recruiting	TCR-T cell for immunotherapy of lung cancer and other solid tumors	30	Advanced lung tumor	Biological: TCR-T cells	Number of patients with dose limiting toxicity	Interventional/Phase1	China	2018/12/16
NCT03979170	Recruiting	Patient-derived organoids of lung cancer to test drug response	50	Lung cancer	Procedure: lung tumor resection	Patient-derived organoid establishment and validation	Observational	Switzerland	2019/4/15
NCT04859166	Recruiting	Prospective primary human lung cancer organoids to predict treatment response	30	Undergo primary surgical resection of a primary lung cancer	Procedure: organoids	Bio-banking, frequency of organoid formation, distribution, proliferation, PDX models of lung cancer, established PDX histologically, established PDX genetically, established PDX biologically, test treatments	Observational	Netherlands	2021/4/15
NCT04951115	Recruiting	A trial with chemotherapy, immunotherapy, and radiotherapy for patients with newly diagnosed stage IV small cell lung cancer	42	ES-SCLC	Drug: carboplatin, cisplatin, etoposide, durvalumab, Radiation: stereotactic body radiotherapy	Therapy toxicities, efficacy of radiation	Interventional/Phase2	United States	2021/6/22
NCT05092009	Recruiting	Lung cancer organoids and patient derived tumor xenografts	600	Undergo primary surgical resection or bronchoscopy or EBUS/EUS-TBNA of a primary lung cancer	Other: tissue and blood	The rate of proliferation and cell death, the size distribution of the organoids, determine the frequency of primary, secondary and tertiary organoid formation	Observational	Netherlands	2021/9/15
NCT05136014	Recruiting	Evaluation of the response to tyrosine kinase inhibitors in localized NSCLC patients with *EGFR* mutation in a patient-derived organoid model	200	NSCLC of any stage undergoing surgical resection	Other: collection of surgical waste	Evaluation of the in vitro efficacy of Osimertinib in a patient-derived organoid model alone or in combination	Observational	France	2021/11/15
NCT05251805	Not recruiting	The safety and feasibility of costal bone marrow aspiration during thoracic surgery	10	Patient undergoing thoracic surgery for a confirmed or suspected lung cancer	Procedure: blood, bone marrow and lung tumor tissue collection	The occurrence of adverse events following costal bone marrow aspiration classified by Calvien-Dindo	Interventional/NA	Belgium	2022/1/27
NCT05332925	Not recruiting	Using ex vivo tumoroids to predict immunotherapy response in NSCLC	25	Patients with advanced/metastatic NSCLC	Drug: standard of care immune checkpoint inhibitors	Feasibility of establishing 3D ex-vivo tumoroid model	Observational	United States	2022/4/10
NCT05411107	Not recruiting	Oral Iloprost for the prevention of lung cancer in former smokers	80	Smokers, Stage I or II lung cancer survivors	Procedure: biospecimen collection, bronchial brush biopsy, bronchoscopy, Drug: Iloprost, placebo administration, Other: questionnaire administration	Change in numerical grade of the worst dysplastic lesion per subject on bronchoscopy	Interventional/Phase2	United States	2022/6/8

PDO: patient-derived organoid, NSCLC: non-small cell lung cancer, ORR: objective response rate, DCR: disease control rate, TCR: T cell receptor, PDX: patient-derived xenograft, ES-SCLS: extensive stage-small cell lung cancer, EBUS-TBNA: endobronchial ultrasound guided transbronchial needle aspiration, EUS: endoscopic ultrasonography. Data is from: https://trialsearch.who.int/ and https://clinicaltrials.gov/

PDO is an important pre-clinical model for determining mechanisms of resistance. Detection of *BRAF* V600E, *KRAS* G12D, *KRAS* G12V, and *PIK3CA* H1047R resistance-associated mutations in long-term cultured Erlotinib-resistant lung cancer PDOs revealed that tumor cells often harbor multiple associated mutations, which means that partial Erlotinib resistant patients require combination therapy to address tumor resistance [57]. The addition of DCLK1 inhibitor DCLK1-IN-1 to the third-generation EGFR-TKI inhibitor Osimertinib resistant PDO downregulates the Wnt/β-catenin signaling pathway, restores tumor sensitivity to Osimertinib [58].

Cisplatin, as the first-line drug for SCLC and NSCLC chemotherapy, inevitably leads to drug resistance during treatment. The mechanism of chemotherapy resistance is complex, and verifying the hypothesis in the new model is necessary [59]. Several compounds were tested in cisplatin-resistant lung cancer PDO to restore chemosensitivity, such as YPN-005, which antagonizes CDK7, Solamargine, which blocks the hedgehog signaling pathway, and Halofuginone, which inhibits PI3K/AKT and MAPK signaling pathways [60–62].

PDO is also an important pre-clinical model for predicting the efficacy of targeted therapies and can be used for prospective adjuvant targeted therapy experiments to reduce treatment costs and improve success rates [63]. In preliminary studies, clinical outcomes were consistent with drug response in PDO models [64]. LUAD PDO carrying *EGFR* and *BRAF* mutations successfully captures the clinical response of the tumor to Dabrafenib/Trametinib combination therapy [4]. The accuracy of PDO in predicting response of patients with *ERBB2* exon 20 insertion and *RET* fusion treated with Poziotinib and Pralsetinib combination is 75% [4]. The high agreement between the PDO drug trial and clinical results promotes Amivantamab as an effective treatment option for NSCLC patients with *EGFR* Exon20ins [65]. Besides, PDO cultured from a patient with *HER2*-A775_G776YVMA insertional mutation summarize the patient’s response to Pyrotinib, as evidenced by subsequent PDX experiments and phase II clinical studies with 53.5% of ORR [37].

Lung cancer PDO can also be used for drug development on neo-cancer targets and large-scale drug screening. As a preclinical model, Lung cancer PDO verifies the anticancer activity of MFF (D) 8–11 peptide mimic, and CKD9 inhibitors SNS032, LY2857785, AZD4573 [66, 67]. Different from traditional organoid culture systems [26, 68], the microfluidic platform developed by Jung and their colleagues and the InSMAR chip designed by Hu and their colleagues can generate PDO with uniform size and load various synthetic or natural anticancer drugs combinations continuously. It is proved that those devices accurately summarize the response of different tumor subtypes and predict the patient’s drug resistance which is highly consistent with PDX and clinical treatment data [9, 69]. Their work reduced the drug susceptibility testing time in PDO to one week. Fast, high-throughput, and accurate, these features improve the feasibility of PDO as a new preclinical model in individualized medicine.

## Challenges and limitations of lung cancer PDO

Establishing pure tumor organoids and maintaining a stable success rate remain significant challenges for PDO application and transformation [11]. Lung cancer organoids culture medium mimics colorectal cancer organoids culture medium, and mismatch of growth factor additives often leads to overgrowth of normal airway organoid (AO) in lung cancer PDO [36]. Although many research groups are actively adjusting the formula to improve the success rate, such as using minimum basic medium or improved medium M26 [23, 27], it is still essential to establish the optimal medium for lung cancer PDO with variable tumor subtypes [70]. B27 supplements, N-acetylcysteine, nicotinamide, SB202190, FGF, and other supplements provide the necessary survival protection signals for cell growth and contribute to organoid formation, but the specific concentrations still need to be adjusted in experiment [70]. Y-27,632, WNT/R-spondin, and EGF may be detrimental to the growth of LUAD PDO [36, 70]. Nutlin-3a and Palbociclib can be used to screen lung cancer cells with TP53 or RB1 mutations from normal organoids [13, 36, 70]. This may contribute to the separation of tumor organoids harbored specific mutation but is not suitable for all samples in PDO purification because lung cancer driver mutations are diverse, and wild-type mutations in normal lung cells [71]. It was also reported that the culture conditions of NSCLC PDO and SCLC PDO were different. R-spondin1 and Wnt3a were important factors for the long-term culture of SCLC tumor organoids [25]. Another strategy for tumor purifying in lung cancer PDO is to remove non-tumor components as much as possible before inoculation. Using biopsy or metastatic tumor tissue to create organoids that lack normal lung epithelium helps to avoid the overgrowth of AO [4, 36]. There are also reports about the removal non-tumor components by suspension culture or separating tumor tissue into single cells in microfluidic systems for PDO formation, but we cannot ignore the importance of extracellular matrix in tumor progression [53, 72]. To establish a reliable organoid model, genomic sequencing combined with morphological observation and marker staining or in vivo tumorigenesis experiments is essential for early identification in lung cancer PDO and clearance of AO [11]. There was no significant difference in the success rate of PDO culture from different lung cancer subtypes [27]. However, the success rate of PDO culture in early lung cancer may be lower than that in advanced lung cancer [12]. Due to different success rates and low repeatability in different research groups [4, 5, 9, 36, 57], we need a standardized methodology. And it would be beneficial to create a database containing the PDO/AO culture system data and histological genetic information [4, 70].

Genomic drifting caused by invitro culturing, which is also affected by differently additives in the medium, is also a major limitation of the PDO models. Presently, organoids are cultured in modified Dulbecco’s modified Eagle’s medium, known as DMEM/F12 medium, with naturally extracted animal ECM matrix as a scaffold. The most widely used ECM is Matrigel extracted from mouse sarcoma [73]. But this natural Matrigel contains more than 1800 proteins, the unknown signals may have selective pressure on tumor growth [74]. This shaping from culture medium may cause specific bias of cell state and affect cell function [75]. Therefore, homogeneity between the PDO models and the primary tumor should be validated by sequencing before further application of this model. In addition, a widely distributed VAF may indicate that lung cancer PDO consist of a heterogeneous population [27], but whether organoids reflect the heterogeneity within tumors needs to be further explored.

## Conclusion and perspective

Lung cancer PDO can reflect the genetic background of tumors better and is easier to culture than PDX, with lower cost and shorter culture period, showing significant advantages and potential in basic and clinical research for lung cancer. It is not only possible to establish PDO from surgical resection specimens but also feasible to establish PDO from malignant pleural effusion or infinitesimal biopsy specimens. The establishment of an optimized culture medium and co-culture system reflects a more realistic TME, which helps us understand the internal mechanism of tumor development and drug resistance. At the same time, large-scale organoid library construction or biobanking improves the use of drug efficacy tests and the credibility of drug screening results, which is expected to provide guidance for individualized medical treatment in the future.

Lung cancer PDO is still facing some problems. Firstly, the success rate of PDO establishment is unstable. The repeatability of organoids between groups has become a limitation of research and application. The referential steps for lung cancer PDO culture have been released, and the standardization protocol needs to be promoted [76, 77]. Secondly, the purity of PDO depends on the sampling, digestion, and culture medium. Further optimization of culture methods and medium components is needed to confirm the applicability for various types of lung cancer [78]. In addition, despite the limited co-culture duration in primary stroma cells and lack of prospective validation for immunotherapy assessment, the co-culture technology of lung cancer PDO should be developed to reconstruct TME in vitro, which will help PDO predict clinical outcomes more accurately and promote the application of organoids in the field of immunotherapy [54].

**Fig. 2:**
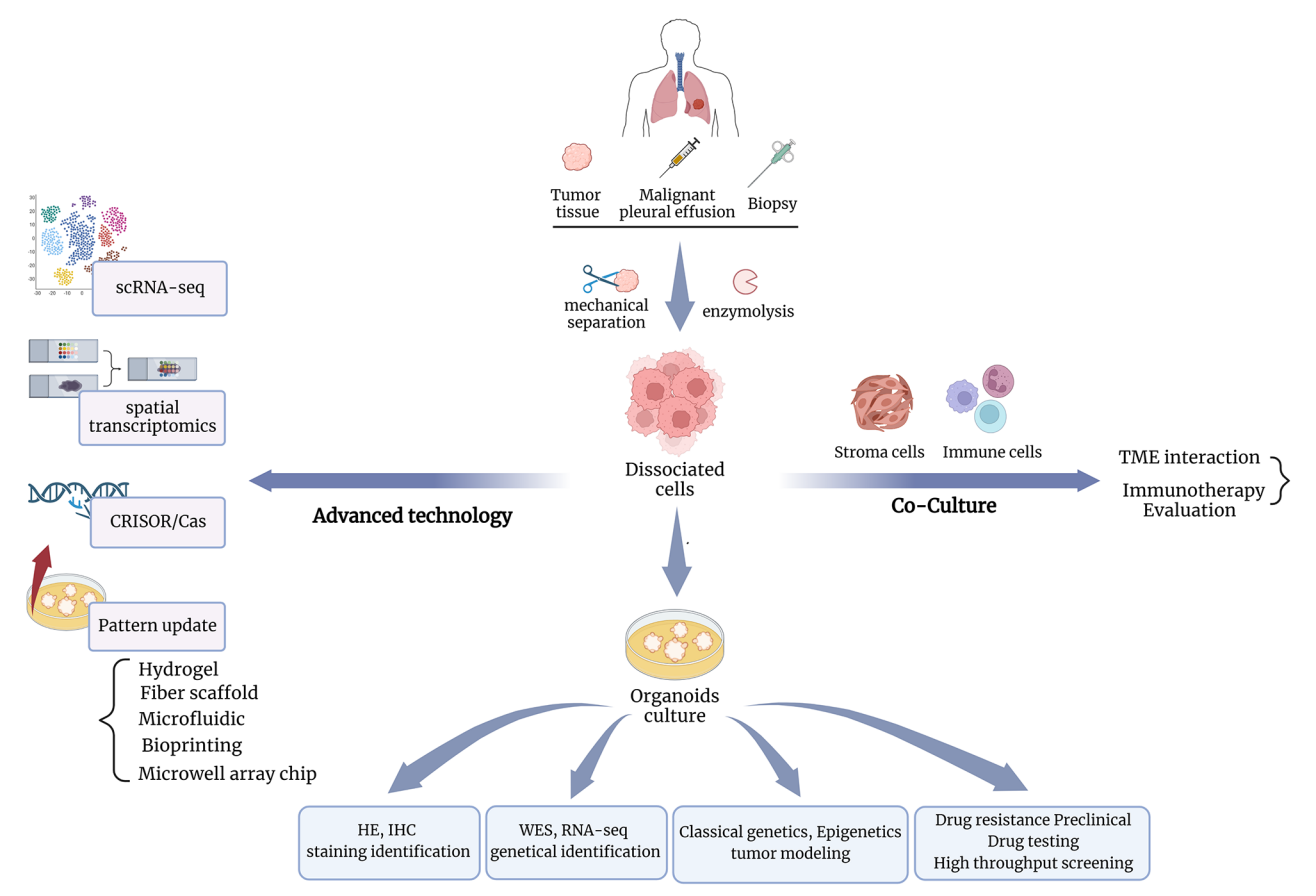
Culture and application of PDO for lung cancer. Surgically excised tissue, biopsies, or malignant pleural fluid from patients are obtained, dissociated to obtain lung cancer tumor cells, and inoculated in special Matrigel-lined media. HE/IHC staining is used for histological identification of the organoid, and WES/RNA-seq assesses the purity of the organoid tumor cells as well as genetic inheritance. Subsequently, organoids can be used for tumor modeling or drug testing. Together with co-culture models, organoids are expected to help further understand the tumor microenvironment of lung cancer and test immunotherapies. With the development of bioengineering technologies, single-cell RNA sequencing, spatial transcriptomics, invitro gene editing, and higher controllable and integrated culture modes will help the application of organoids

With the rapid development of biomedical engineering, the advanced technology that has been applied to other tumor organoids can be introduced into the culture of lung cancer PDO, and the development prospect will be extensive [79] (Fig. 2).

1. Single-cell RNA-sequencing (scRNA-seq) is one of the most accurate tools to verify whether PDO truly generalizes the characteristics and heterogeneity of primary tumors. The scRNA-seq allows analysis of tumor cell genomes, transcriptomes, and epigenomes at the single-cell level, tracking the lineage of cells, which helps with the development of precision oncology [80]. The upgrade from RNA-seq to scRNA-seq is expected to match the unique clinical and biological characteristics of patients with the best treatment combination, thereby maximizing clinical benefits. Spatial transcriptomics is a rapidly developing method which allows preserving crucial spatial context of regulatory processes. Integration of spatial and single-cell transcriptomic data provide a comprehensive cell resolution spatial map for morphogenesis research and tumor tissue architecture analysis [81, 82].
2. Genetic engineering enables organoids to study the role of interested gene mutations in cancer and develop corresponding anticancer therapies. Dox-induced HER2-overexpressing conditioned cells were generated from iPSC cells using the CRISPR/Cas9 genome editing system, and corresponding organoids with typical AAH characteristics were successfully cultured. CRISPR/Cas9 allows researchers to build specific organoids to explore vital evolutionary processes in early-stage lung cancer [24, 83]. Interestingly, the induction of mature lung organoids from iPSC requires the co-culture of human fetal fibroblasts to support the differentiation of stem cells into alveoli [24]. Besides, CRISPR-HOT, developed by Artegiani and their colleagues, enables efficient and visual gene editing in organoids [84]. Rare and specific subsets are often observed in lung cancer [85]. Genome-Wide CRISPR/Cas9 screen for rare and interesting genes in organoids will significantly promote the development of tumor research [86].
3. Advanced engineering methods are complementing the traditional organoid culture model. Mechanically adjustable biomaterials such as hydrogels and fibrous scaffolds are being developed to replace traditional ECM. At the same time, the use of microporous arrays, 3D bioprinting, and microfluidics in organoids realizes high-throughput drug testing, immunotherapy screening, and vascularization in organoids [87]. Lung cancer PDO-related microchips and microfluidic platforms have been developed to enable large-scale drug testing within a week [9, 69]. The researchers also developed an automatic IF multiplexing for FFPE sections from lung cancer PDO to detect the expression of multiple critical biomarkers on a single slide [88]. With machine learning, SigMaps can integrate and generate molecular interactions of specific tumor-associated proteins in lung cancer and perform large-scale validation of predicted proteins in PDO [89].

In conclusion, as an emerging preclinical model, lung cancer PDO has excellent potential and broad prospects in the research and treatment of lung cancer. Although there are still some challenges, the rapid development of culture technology will bring greater possibilities for the application of lung cancer PDO in clinical treatment guidelines.

## Data Availability

Not applicable.

## References

[1] Sung H, Ferlay J, Siegel RL, Laversanne M, Soerjomataram I, Jemal A, Bray F (2021). Global Cancer Statistics 2020: GLOBOCAN estimates of incidence and Mortality Worldwide for 36 cancers in 185 countries. CA. Cancer J. Clin.

[2] Herbst RS, Morgensztern D, Boshoff C (2018). The biology and management of non-small cell lung cancer. Nature.

[3] Elbadawy M, Usui T, Mori T, Tsunedomi R, Hazama S, Nabeta R, Uchide T, Fukushima R, Yoshida T, Shibutani M, Tanaka T, Masuda S, Okada R, Ichikawa R, Omatsu T, Mizutani T, Katayama Y, Noguchi S, Iwai S, Nakagawa T, Shinohara Y, Kaneda M, Yamawaki H, Sasaki K (2019). Establishment of a novel experimental model for muscle-invasive bladder cancer using a dog bladder cancer organoid culture. Cancer Sci.

[4] Kim SY, Kim SM, Lim S, Lee JY, Choi SJ, Yang SD, Yun MR, Kim CG, Gu SR, Park C, Park AY, Lim SM, Heo SG, Kim H (2021). Cho, modeling clinical responses to targeted therapies by patient-derived organoids of Advanced Lung Adenocarcinoma. Clin. Cancer Res.

[5] Z. Li, Y. Qian, W. Li, L. Liu, L. Yu, X. Liu, G. Wu, Y. Wang, W. Luo, F. Fang, Y. Liu, F. Song, Z. Cai, W. Chen, W. Huang, Human lung adenocarcinoma-derived organoid models for drug screening. *iScience* **23**, 101411 (2020) 10.1016/j.isci.2020.101411 PMC7415928 32771979

[6] Tuveson D, Clevers H (2019). Cancer modeling meets human organoid technology. Science.

[7] Elbadawy M, Sato Y, Mori T, Goto Y, Hayashi K, Yamanaka M, Azakami D, Uchide T, Fukushima R, Yoshida T, Shibutani M, Kobayashi M, Shinohara Y, Abugomaa A, Kaneda M, Yamawaki H, Usui T, Sasaki K (2021). Anti-tumor effect of trametinib in bladder cancer organoid and the underlying mechanism. Cancer Biol. Ther.

[8] Chen F, Liu J, Flight RM, Naughton KJ, Lukyanchuk A, Edgin AR, Song X, Zhang H, Wong KK, Moseley HNB, Wang C, Brainson CF (2021). Cellular Origins of EGFR-Driven Lung Cancer cells determine sensitivity to Therapy. Adv. Sci.

[9] Hu Y, Sui X, Song F, Li Y, Li K, Chen Z, Yang F, Chen X, Zhang Y, Wang X, Liu Q, Li C, Zou B, Chen X, Wang J, Liu P (2021). Lung cancer organoids analyzed on microwell arrays predict drug responses of patients within a week. Nat. Commun.

[10] Takahashi N, Hoshi H, Higa A, Hiyama G, Tamura H, Ogawa M, Takagi K, Goda K, Okabe N, Muto S, Suzuki H, Shimomura K, Watanabe S, Takagi M (2019). An in Vitro System for evaluating molecular targeted drugs using lung patient-derived Tumor Organoids. Cells.

[11] Dijkstra KK, Monkhorst K, Schipper LJ, Hartemink KJ, Smit EF, Kaing S, de Groot R, Wolkers MC, Clevers H, Cuppen E (2020). Voest, Challenges in establishing pure Lung Cancer Organoids Limit their utility for Personalized Medicine. Cell Rep.

[12] Della Corte CM, Barra G, Ciaramella V, Di Liello R, Vicidomini G, Zappavigna S, Luce A, Abate M, Fiorelli A, Caraglia M, Santini M, Martinelli E, Troiani T, Ciardiello F, Morgillo F (2019). Antitumor activity of dual blockade of PD-L1 and MEK in NSCLC patients derived three-dimensional spheroid cultures. J. Exp. Clin. Cancer Res.

[13] K.K. Dijkstra, C.M. Cattaneo, F. Weeber, M. Chalabi, J. van de Haar, L.F. Fanchi, M. Slagter, D.L. van der Velden, S. Kaing, S. Kelderman, N. van Rooij, M.E. van Leerdam, A. Depla, E.F. Smit, K.J. Hartemink, R. de Groot, M.C. Wolkers, N. Sachs, P. Snaebjornsson, K. Monkhorst, J. Haanen, H. Clevers, T.N. Schumacher, E. E. Voest, Generation of tumor-reactive T cells by co-culture of peripheral blood lymphocytes and tumor organoids. Cell **174**, 1586–1598 e1512 (2018) 10.1016/j.cell.2018.07.009 PMC6558289 30100188

[14] Zheng C, Sun YH, Ye XL, Chen HQ, Ji HB (2011). Establishment and characterization of primary lung cancer cell lines from chinese population. Acta Pharmacol. Sin.

[15] Gazdar AF, Gao B, Minna JD (2010). Lung cancer cell lines: useless artifacts or invaluable tools for medical science?. Lung Cancer.

[16] Hutmacher DW (2010). Biomaterials offer cancer research the third dimension. Nat. Mater.

[17] Ben-David U, Siranosian B, Ha G, Tang H, Oren Y, Hinohara K, Strathdee CA, Dempster J, Lyons NJ, Burns R, Nag A, Kugener G, Cimini B, Tsvetkov P, Maruvka YE, O’Rourke R, Garrity A, Tubelli AA, Bandopadhayay P, Tsherniak A, Vazquez F, Wong B, Birger C, Ghandi M, Thorner AR, Bittker JA, Meyerson M, Getz G, Beroukhim R, Golub TR (2018). Genetic and transcriptional evolution alters cancer cell line drug response. Nature.

[18] Wilding JL, Bodmer WF (2014). Cancer cell lines for drug discovery and development. Cancer Res.

[19] Yoshida GJ (2020). Applications of patient-derived tumor xenograft models and tumor organoids. J. Hematol. Oncol.

[20] Meraz IM, Majidi M, Meng F, Shao R, Ha MJ, Neri S, Fang B, Lin SH, Tinkey PT, Shpall EJ, Morris J, Roth JA (2019). An Improved patient-derived xenograft humanized mouse model for evaluation of Lung Cancer Immune responses. Cancer Immunol. Res.

[21] Abdolahi S, Ghazvinian Z, Muhammadnejad S, Saleh M, Asadzadeh Aghdaei H, Baghaei K (2022). Patient-derived xenograft (PDX) models, applications and challenges in cancer research. J. Transl Med.

[22] Junttila MR, de Sauvage FJ (2013). Influence of tumour micro-environment heterogeneity on therapeutic response. Nature.

[23] Shi R, Radulovich N, Ng C, Liu N, Notsuda H, Cabanero M, Martins-Filho SN, Raghavan V, Li Q, Mer AS, Rosen JC, Li M, Wang YH, Tamblyn L, Pham NA, Haibe-Kains B, Liu G, Moghal N, Tsao MS (2020). Organoid cultures as preclinical models of Non-Small Cell Lung Cancer. Clin. Cancer Res.

[24] Miura A, Yamada D, Nakamura M, Tomida S, Shimizu D, Jiang Y, Takao T, Yamamoto H, Suzawa K, Shien K, Yamane M, Sakaguchi M, Toyooka S, Takarada T (2021). Oncogenic potential of human pluripotent stem cell-derived lung organoids with HER2 overexpression. Int. J. Cancer.

[25] Choi SY, Cho YH, Kim DS, Ji W, Choi CM, Lee JC, Rho JK, Jeong GS (2021). Establishment and long-term expansion of small cell Lung Cancer patient-derived Tumor Organoids. Int. J. Mol. Sci.

[26] Chen JH, Chu XP, Zhang JT, Nie Q, Tang WF, Su J, Yan HH, Zheng HP, Chen ZX, Chen X, Song MM, Yi X, Li PS, Guan YF, Li G, Deng CX, Rosell R, Wu YL (2020). Zhong, genomic characteristics and drug screening among organoids derived from non-small cell lung cancer patients. Thorac. Cancer.

[27] Kim M, Mun H, Sung CO, Cho EJ, Jeon HJ, Chun SM, Jung DJ, Shin TH, Jeong GS, Kim DK, Choi EK, Jeong SY, Taylor AM, Jain S, Meyerson M, Jang SJ (2019). Patient-derived lung cancer organoids as in vitro cancer models for therapeutic screening. Nat. Commun.

[28] P.H. Chen, L. Cai, K. Huffman, C. Yang, J. Kim, B. Faubert, L. Boroughs, B. Ko, J. Sudderth, E.A. McMillan, L. Girard, D. Chen, M. Peyton, M.D. Shields, B. Yao, D.S. Shames, H.S. Kim, B. Timmons, I. Sekine, R. Britt, S. Weber, L.A. Byers, J.V. Heymach, J. Chen, M.A. White, J.D. Minna, G. Xiao, R.J. DeBerardinis, Metabolic Diversity in Human Non-Small Cell Lung Cancer Cells. Mol. Cell **76**, 838–851 e835 (2019) 10.1016/j.molcel.2019.08.028 PMC6898782 31564558

[29] Cox AG, Hwang KL, Brown KK, Evason K, Beltz S, Tsomides A, O’Connor K, Galli GG, Yimlamai D, Chhangawala S, Yuan M, Lien EC, Wucherpfennig J, Nissim S, Minami A, Cohen DE, Camargo FD, Asara JM, Houvras Y, Stainier DYR, Goessling W (2016). Yap reprograms glutamine metabolism to increase nucleotide biosynthesis and enable liver growth. Nat. Cell. Biol.

[30] Zhao JS, Shi S, Qu HY, Keckesova Z, Cao ZJ, Yang LX, Yu X, Feng L, Shi Z, Krakowiak J, Mao RY, Shen YT, Fan YM, Fu TM, Ye C, Xu D, Gao X, You J, Li W, Liang T, Lu Z, Feng YX (2022). Glutamine synthetase licenses APC/C-mediated mitotic progression to drive cell growth. Nat. Metab.

[31] Chen B, Song Y, Zhan Y, Zhou S, Ke J, Ao W, Zhang Y, Liang Q, He M, Li S, Xie F, Huang H, Chan WN, Cheung AHK, Ma BBY, Kang W, To KF, Xiao J (2022). Fangchinoline inhibits non-small cell lung cancer metastasis by reversing epithelial-mesenchymal transition and suppressing the cytosolic ROS-related Akt-mTOR signaling pathway. Cancer Lett.

[32] Windmoller BA, Beshay M, Helweg LP, Flottmann C, Beermann M, Forster C, Wilkens L, Greiner JFW, Kaltschmidt C, Kaltschmidt B (2021). Novel Primary Human Cancer Stem-Like Cell populations from Non-Small Cell Lung Cancer: inhibition of cell survival by targeting NF-kappaB and MYC signaling. Cells.

[33] Taverna JA, Hung CN, DeArmond DT, Chen M, Lin CL, Osmulski PA, Gaczynska ME, Wang CM, Lucio ND, Chou CW, Chen CL, Nazarullah A, Lampkin SR, Qiu L, Bearss DJ, Warner S, Whatcott CJ, Mouritsen L, Wade M, Weitman S, Mesa RA, Kirma NB, Chao WT (2020). Huang, single-cell proteomic profiling identifies combined AXL and JAK1 Inhibition as a Novel Therapeutic Strategy for Lung Cancer. Cancer Res.

[34] Rowbotham SP, Li F, Dost AFM, Louie SM, Marsh BP, Pessina P, Anbarasu CR, Brainson CF, Tuminello SJ, Lieberman A, Ryeom S, Schlaeger TM, Aronow BJ, Watanabe H, Wong KK, Kim CF (2018). H3K9 methyltransferases and demethylases control lung tumor-propagating cells and lung cancer progression. Nat. Commun.

[35] Marshall LJ, Triunfol M, Seidle T (2020). Patient-derived xenograft vs. organoids: a preliminary analysis of Cancer Research output, funding and Human Health Impact in 2014–2019. Animals.

[36] Sachs N, Papaspyropoulos A, Zomer-van Ommen DD, Heo I, Bottinger L, Klay D, Weeber F, Huelsz-Prince G, Iakobachvili N, Amatngalim GD, de Ligt J, van Hoeck A, Proost N, Viveen MC, Lyubimova A, Teeven L, Derakhshan S, Korving J, Begthel H, Dekkers JF, Kumawat K, Ramos E, van Oosterhout MF, Offerhaus GJ, Wiener DJ, Olimpio EP, Dijkstra KK, Smit EF, van der Linden M, Jaksani S, van de Ven M, Jonkers J, Rios AC, Voest EE, van Moorsel CH, van der Ent CK, Cuppen E, van Oudenaarden A, Coenjaerts FE, Meyaard L, Bont LJ, Peters PJ, Tans SJ, van Zon JS, Boj SF, Vries RG, Beekman JM, Clevers H (2019). Long-term expanding human airway organoids for disease modeling. Embo J.

[37] Wang Y, Jiang T, Qin Z, Jiang J, Wang Q, Yang S, Rivard C, Gao G, Ng TL, Tu MM, Yu H, Ji H, Zhou C, Ren S, Zhang J, Bunn P, Doebele RC, Camidge DR, Hirsch FR (2019). HER2 exon 20 insertions in non-small-cell lung cancer are sensitive to the irreversible pan-HER receptor tyrosine kinase inhibitor pyrotinib. Ann. Oncol.

[38] Zeng Z, Li J, Zhang J, Li Y, Liu X, Chen J, Huang Z, Wu Q, Gong Y, Xie C (2021). Immune and stromal scoring system associated with tumor microenvironment and prognosis: a gene-based multi-cancer analysis. J. Transl Med.

[39] Lambrechts D, Wauters E, Boeckx B, Aibar S, Nittner D, Burton O, Bassez A, Decaluwe H, Pircher A, Van den Eynde K, Weynand B, Verbeken E, De Leyn P, Liston A, Vansteenkiste J, Carmeliet P, Aerts S, Thienpont B (2018). Phenotype molding of stromal cells in the lung tumor microenvironment. Nat. Med.

[40] Quail DF, Joyce JA (2013). Microenvironmental regulation of tumor progression and metastasis. Nat. Med.

[41] Irie H, Ozaki M, Chubachi S, Hegab AE, Tsutsumi A, Kameyama N, Sakurai K, Nakayama S, Kagawa S, Wada S, Ishii M, Betsuyaku T, Fukunaga K (2022). Short-term intermittent cigarette smoke exposure enhances alveolar type 2 cell stemness via fatty acid oxidation. Respir Res.

[42] Rebelo SP, Pinto C, Martins TR, Harrer N, Estrada MF, Loza-Alvarez P, Cabecadas J, Alves PM, Gualda EJ, Sommergruber W, Brito C (2018). 3D-3-culture: a tool to unveil macrophage plasticity in the tumour microenvironment. Biomaterials.

[43] Majety M, Pradel LP, Gies M, Ries CH (2015). Fibroblasts influence survival and therapeutic response in a 3D co-culture model. PLoS One.

[44] Sandor GO, Soos AA, Lorincz P, Rojko L, Harko T, Bogyo L, Tolgyes T, Bursics A, Buzas EI, Moldvay J, Wiener Z (2021). Wnt activity and cell proliferation are coupled to Extracellular Vesicle Release in multiple organoid models. Front. Cell. Dev. Biol.

[45] S. Chen, A. Giannakou, J. Golas, K.G. Geles, Multidimensional coculture system to model lung squamous carcinoma progression. J. Vis. Exp., e60644 (2020) 10.3791/60644 32250351

[46] Kozlowski MT, Crook CJ, Ku HT (2021). Towards organoid culture without Matrigel. Commun. Biol.

[47] Del Bufalo F, Manzo T, Hoyos V, Yagyu S, Caruana I, Jacot J, Benavides O, Rosen D (2016). Brenner, 3D modeling of human cancer: a PEG-fibrin hydrogel system to study the role of tumor microenvironment and recapitulate the in vivo effect of oncolytic adenovirus. Biomaterials.

[48] Borghaei H, Paz-Ares L, Horn L, Spigel DR, Steins M, Ready NE, Chow LQ, Vokes EE, Felip E, Holgado E, Barlesi F, Kohlhaufl M, Arrieta O, Burgio MA, Fayette J, Lena H, Poddubskaya E, Gerber DE, Gettinger SN, Rudin CM, Rizvi N, Crino L, Blumenschein GR, Antonia SJ, Dorange C, Harbison CT, Graf Finckenstein F, Brahmer JR (2015). Nivolumab versus Docetaxel in Advanced Nonsquamous Non-Small-Cell Lung Cancer. N Engl. J. Med.

[49] Garon EB, Rizvi NA, Hui R, Leighl N, Balmanoukian AS, Eder JP, Patnaik A, Aggarwal C, Gubens M, Horn L, Carcereny E, Ahn MJ, Felip E, Lee JS, Hellmann MD, Hamid O, Goldman JW, Soria JC, Dolled-Filhart M, Rutledge RZ, Zhang J, Lunceford JK, Rangwala R, Lubiniecki GM, Roach C, Emancipator K, Gandhi L (2015). Investigators, Pembrolizumab for the treatment of non-small-cell lung cancer. N Engl. J. Med.

[50] Schoenfeld AJ, Hellmann MD (2020). Acquired Resistance to Immune Checkpoint inhibitors. Cancer Cell.

[51] Cattaneo CM, Dijkstra KK, Fanchi LF, Kelderman S, Kaing S, van Rooij N, van den Brink S, Schumacher TN (2020). Voest, Tumor organoid-T-cell coculture systems. Nat. Protoc.

[52] Cerignoli F, Abassi YA, Lamarche BJ, Guenther G, Santa Ana D, Guimet D, Zhang W, Zhang J, Xi B (2018). In vitro immunotherapy potency assays using real-time cell analysis. PLoS One.

[53] S. Ding, C. Hsu, Z. Wang, N.R. Natesh, R. Millen, M. Negrete, N. Giroux, G.O. Rivera, A. Dohlman, S. Bose, T. Rotstein, K. Spiller, A. Yeung, Z. Sun, C. Jiang, R. Xi, B. Wilkin, P.M. Randon, I. Williamson, D.A. Nelson, D. Delubac, S. Oh, G. Rupprecht, J. Isaacs, J. Jia, C. Chen, J.P. Shen, S. Kopetz, S. McCall, A. Smith, N. Gjorevski, A.C. Walz, S. Antonia, E. Marrer-Berger, H. Clevers, D. Hsu, X. Shen, Patient-derived micro-organospheres enable clinical precision oncology. Cell Stem Cell **29**, 905–917 e906 (2022) 10.1016/j.stem.2022.04.006 PMC9177814 35508177

[54] J.T. Neal, X. Li, J. Zhu, V. Giangarra, C.L. Grzeskowiak, J. Ju, I.H. Liu, S.H. Chiou, A.A. Salahudeen, A.R. Smith, B.C. Deutsch, L. Liao, A.J. Zemek, F. Zhao, K. Karlsson, L.M. Schultz, T.J. Metzner, L.D. Nadauld, Y.Y. Tseng, S. Alkhairy, C. Oh, P. Keskula, D. Mendoza-Villanueva, F.M. De La Vega, P.L. Kunz, J.C. Liao, J.T. Leppert, J.B. Sunwoo, C. Sabatti, J.S. Boehm, W.C. Hahn, G.X.Y. Zheng, M.M. Davis, C.J. Kuo, Organoid modeling of the tumor immune microenvironment. Cell **175**, 1972–1988 e1916 (2018) 10.1016/j.cell.2018.11.021 PMC6656687 30550791

[55] Li H, Harrison EB, Li H, Hirabayashi K, Chen J, Li QX, Gunn J, Weiss J, Savoldo B, Parker JS, Pecot CV, Dotti G, Du H (2022). Targeting brain lesions of non-small cell lung cancer by enhancing CCL2-mediated CAR-T cell migration. Nat. Commun.

[56] Bironzo P, Primo L, Novello S, Righi L, Candeloro S, Manganaro L, Bussolino F, Pirri F, Scagliotti GV (2022). Clinical-molecular prospective cohort study in Non-Small Cell Lung Cancer (PROMOLE study): a Comprehensive Approach to identify new predictive markers of pharmacological response. Clin. Lung Cancer.

[57] Banda M, McKim KL, Myers MB, Inoue M, Parsons BL (2020). Outgrowth of erlotinib-resistant subpopulations recapitulated in patient-derived lung tumor spheroids and organoids. PLoS One.

[58] Yan R, Fan X, Xiao Z, Liu H, Huang X, Liu J, Zhang S, Yao J, An G, Ge Y (2022). Inhibition of DCLK1 sensitizes resistant lung adenocarcinomas to EGFR-TKI through suppression of Wnt/beta-Catenin activity and cancer stemness. Cancer Lett.

[59] Herzog BH, Devarakonda S, Govindan R (2021). Overcoming Chemotherapy Resistance in SCLC. J. Thorac. Oncol.

[60] Han Y, Shi J, Xu Z, Zhang Y, Cao X, Yu J, Li J, Xu S (2022). Identification of solamargine as a cisplatin sensitizer through phenotypical screening in cisplatin-resistant NSCLC organoids. Front. Pharmacol.

[61] Li H, Zhang Y, Lan X, Yu J, Yang C, Sun Z, Kang P, Han Y, Yu D (2021). Halofuginone sensitizes Lung Cancer Organoids to Cisplatin via suppressing PI3K/AKT and MAPK signaling pathways. Front. Cell. Dev. Biol.

[62] Choi YJ, Lee H, Kim DS, Kim DH, Kang MH, Cho YH, Choi CM, Yoo J, Lee KO, Choi EK, Lee JC, Rho JK (2021). Discovery of a novel CDK7 inhibitor YPN-005 in small cell lung cancer. Eur. J. Pharmacol.

[63] Bie Y, Wang J, Xiong L, Wang D, Liao J, Zhang Y, Lin H (2021). Lung adenocarcinoma organoids harboring EGFR 19Del and L643V double mutations respond to osimertinib and gefitinib: a case report. Med. (Baltim).

[64] Peng KC, Su JW, Xie Z, Wang HM, Fang MM, Li WF, Chen YQ, Guan XH, Su J, Yan HH, Zhang XC, Tu HY, Zhou Q, Chen HJ, Wu YL, Yang JJ (2022). Clinical outcomes of EGFR+/METamp + vs. EGFR+/METamp- untreated patients with advanced non-small cell lung cancer. Thorac. Cancer.

[65] Yun J, Lee SH, Kim SY, Jeong SY, Kim JH, Pyo KH, Park CW, Heo SG, Yun MR, Lim S, Lim SM, Hong MH, Kim HR, Thayu M, Curtin JC, Knoblauch RE, Lorenzi MV, Roshak A, Cho BC (2020). Antitumor Activity of Amivantamab (JNJ-61186372), an EGFR-MET bispecific antibody, in Diverse Models of EGFR exon 20 insertion-driven NSCLC. Cancer Discov.

[66] Seo JH, Chae YC, Kossenkov AV, Lee YG, Tang HY, Agarwal E, Gabrilovich DI, Languino LR, Speicher DW, Shastrula PK, Storaci AM, Ferrero S, Gaudioso G, Caroli M, Tosi D, Giroda M, Vaira V, Rebecca VW, Herlyn M, Xiao M, Fingerman D, Martorella A, Skordalakes E, Altieri DC (2019). MFF Regulation of mitochondrial cell death is a therapeutic target in Cancer. Cancer Res.

[67] Padmanabhan J, Saha B, Powell C, Mo Q, Perez BA, Chellappan S (2021). Inhibitors targeting CDK9 Show High Efficacy against Osimertinib and AMG510 resistant lung adenocarcinoma cells. Cancers.

[68] Li YF, Gao Y, Liang BW, Cao XQ, Sun ZJ, Yu JH, Liu ZD, Han Y (2020). Patient-derived organoids of non-small cells lung cancer and their application for drug screening. Neoplasma.

[69] Jung DJ, Shin TH, Kim M, Sung CO, Jang SJ, Jeong GS (2019). A one-stop microfluidic-based lung cancer organoid culture platform for testing drug sensitivity. Lab. Chip.

[70] Ma HC, Zhu YJ, Zhou R, Yu YY, Xiao ZZ, Zhang HB (2022). Lung cancer organoids, a promising model still with long way to go. Crit. Rev. Oncol. Hematol.

[71] Gao W, Mady HH, Melhem MF, Keohavong P (2009). Analysis of p53 mutations in histologically normal lung tissues and lung tumors from non-small cell lung cancer patients. Mol. Carcinog.

[72] Tamura H, Higa A, Hoshi H, Hiyama G, Takahashi N, Ryufuku M, Morisawa G, Yanagisawa Y, Ito E, Imai JI, Dobashi Y, Katahira K, Soeda S, Watanabe T, Fujimori K, Watanabe S, Takagi M (2018). Evaluation of anticancer agents using patient-derived tumor organoids characteristically similar to source tissues. Oncol. Rep.

[73] Orkin RW, Gehron P, McGoodwin EB, Martin GR, Valentine T, Swarm R (1977). A murine tumor producing a matrix of basement membrane. J. Exp. Med.

[74] Hughes CS, Postovit LM, Lajoie GA (2010). Matrigel: a complex protein mixture required for optimal growth of cell culture. Proteomics.

[75] S. Raghavan, P.S. Winter, A.W. Navia, H.L. Williams, A. DenAdel, K.E. Lowder, J. Galvez-Reyes, R.L. Kalekar, N. Mulugeta, K.S. Kapner, M.S. Raghavan, A.A. Borah, N. Liu, S.A. Vayrynen, A.D. Costa, R.W.S. Ng, J. Wang, E.K. Hill, D.Y. Ragon, L.K. Brais, A.M. Jaeger, L.F. Spurr, Y.Y. Li, A.D. Cherniack, M.A. Booker, E.F. Cohen, M.Y. Tolstorukov, I. Wakiro, A. Rotem, B.E. Johnson, J.M. McFarland, E.T. Sicinska, T.E. Jacks, R.J. Sullivan, G.I. Shapiro, T.E. Clancy, K. Perez, D.A. Rubinson, K. Ng, J.M. Cleary, L. Crawford, S.R. Manalis, J.A. Nowak, B.M. Wolpin, W.C. Hahn, A. J. Aguirre, A. K. Shalek, Microenvironment drives cell state, plasticity, and drug response in pancreatic cancer. Cell **184**, 6119–6137 e6126 (2021) 10.1016/j.cell.2021.11.017 PMC8822455 34890551

[76] D. Barbosa Rabago, C.M. Blakely, F. Haderk, T.G. Bivona, Profiling sensitivity to targeted therapies in EGFR-mutant NSCLC patient-derived organoids. J. Vis. Exp., e63039 (2021) 10.3791/63039 34866626

[77] Li Z, Yu L, Chen D, Meng Z, Chen W, Huang W (2021). Protocol for generation of lung adenocarcinoma organoids from clinical samples. STAR. Protoc.

[78] Lee D, Kim Y, Chung C (2021). Scientific validation and clinical application of Lung Cancer Organoids. Cells.

[79] Qu J, Kalyani FS, Liu L, Cheng T, Chen L (2021). Tumor organoids: synergistic applications, current challenges, and future prospects in cancer therapy. Cancer Commun.

[80] Tanay A, Regev A (2017). Scaling single-cell genomics from phenomenology to mechanism. Nature.

[81] Lohoff T, Ghazanfar S, Missarova A, Koulena N, Pierson N, Griffiths JA, Bardot ES, Eng CL, Tyser RCV, Argelaguet R, Guibentif C, Srinivas S, Briscoe J, Simons BD, Hadjantonakis AK, Gottgens B, Reik W, Nichols J, Cai L, Marioni JC (2022). Integration of spatial and single-cell transcriptomic data elucidates mouse organogenesis. Nat. Biotechnol.

[82] Moncada R, Barkley D, Wagner F, Chiodin M, Devlin JC, Baron M, Hajdu CH, Simeone DM, Yanai I (2020). Integrating microarray-based spatial transcriptomics and single-cell RNA-seq reveals tissue architecture in pancreatic ductal adenocarcinomas. Nat. Biotechnol.

[83] A.F.M. Dost, A.L. Moye, M. Vedaie, L.M. Tran, E. Fung, D. Heinze, C. Villacorta-Martin, J. Huang, R. Hekman, J.H. Kwan, B.C. Blum, S.M. Louie, S.P. Rowbotham, J. Sainz de Aja, M.E. Piper, P.J. Bhetariya, R.T. Bronson, A. Emili, G. Mostoslavsky, G.A. Fishbein, W.D. Wallace, K. Krysan, S.M. Dubinett, J. Yanagawa, D.N. Kotton, C.F. Kim, Organoids model transcriptional hallmarks of oncogenic KRAS activation in lung epithelial progenitor cells. Cell Stem Cell **27**, 663–678 e668 (2020) 10.1016/j.stem.2020.07.022 PMC7541765 32891189

[84] Artegiani B, Hendriks D, Beumer J, Kok R, Zheng X, Joore I, Chuva de Sousa Lopes S, van Zon J, Tans S, Clevers H (2020). Fast and efficient generation of knock-in human organoids using homology-independent CRISPR-Cas9 precision genome editing. Nat. Cell. Biol.

[85] J. Chen, H. Yang, A.S.M. Teo, L.B. Amer, F.G. Sherbaf, C.Q. Tan, J.J.S. Alvarez, B. Lu, J.Q. Lim, A. Takano, R. Nahar, Y.Y. Lee, C.Z.J. Phua, K.P. Chua, L. Suteja, P.J. Chen, M.M. Chang, T.P.T. Koh, B.H. Ong, D. Anantham, A.A.L. Hsu, A. Gogna, C.W. Too, Z.W. Aung, Y.F. Lee, L. Wang, T.K.H. Lim, A. Wilm, P.S. Choi, P.Y. Ng, C.K. Toh, W.T. Lim, S. Ma, B. Lim, J. Liu, W.L. Tam, A.J. Skanderup, J.P.S. Yeong, E.H. Tan, C.L. Creasy, D. S. W. A.M. Tan, Hillmer, W. Zhai, Genomic landscape of lung adenocarcinoma in East Asians. Nature Genet **52**, 177–186 (2020) 10.1038/s41588-019-0569-6 32015526

[86] Liang J, Zhao H, Diplas BH, Liu S, Liu J, Wang D, Lu Y, Zhu Q, Wu J, Wang W, Yan H, Zeng YX, Wang X, Jiao Y (2020). Genome-wide CRISPR-Cas9 screen reveals selective vulnerability of ATRX-Mutant cancers to WEE1 inhibition. Cancer Res.

[87] Luo Z, Zhou X, Mandal K, He N, Wennerberg W, Qu M, Jiang X, Sun W, Khademhosseini A (2021). Reconstructing the tumor architecture into organoids. Adv. Drug Deliv Rev.

[88] Bhaumik S, Boyer J, Banerjee C, Clark S, Sebastiao N, Vela E, Towne P (2020). Fluorescent multiplexing of 3D spheroids: analysis of biomarkers using automated immunohistochemistry staining platform and multispectral imaging. J. Cell. Biochem.

[89] Broyde J, Simpson DR, Murray D, Paull EO, Chu BW, Tagore S, Jones SJ, Griffin AT, Giorgi FM, Lachmann A, Jackson P, Sweet-Cordero EA, Honig B, Califano A (2021). Oncoprotein-specific molecular interaction maps (SigMaps) for cancer network analyses. Nat. Biotechnol.

